# Seizures Beyond the Heart: Unveiling Neurocysticercosis After Cardiac Surgery

**DOI:** 10.7759/cureus.100259

**Published:** 2025-12-28

**Authors:** Vijay Adabala, Kalicharan Das, Jameel A Aleem, Anik Goel, Swati Pathak

**Affiliations:** 1 Anaesthesiology, All India Institute of Medical Sciences, Rae Bareli, Raebareli, IND; 2 Anaesthesiology and Critical Care, All India Institute of Medical Sciences, Rae Bareli, Raebareli, IND; 3 Cardiacvascular Thoracic Surgery, All India Institute of Medical Sciences, Rae Bareli, Raebareli, IND

**Keywords:** computerized tomography, epileptic seizures, mitral valve replacement, neurocystecercosis, post-operative outcome in cardiac surgery

## Abstract

Postoperative seizures are uncommon but clinically significant complications after cardiac surgery, usually attributed to hypoxia, metabolic disturbances, embolic events, or stroke. In endemic regions, such as India, however, neurocysticercosis (NCC) remains a leading cause of seizures and may present incidentally in the perioperative period. We report the case of a 39-year-old woman who underwent elective mitral valve repair and developed new-onset generalized seizures in the immediate postoperative phase. Urgent neuroimaging revealed a calcified parietal lesion consistent with old NCC. Seizure control was achieved with antiepileptic therapy, and the patient recovered uneventfully. This case emphasizes the need to consider NCC in the differential diagnosis of postoperative seizures, particularly in endemic populations, and highlights the importance of timely imaging and supportive management in optimizing outcomes.

## Introduction

Postoperative seizures are recognized but relatively uncommon complications following cardiac surgery [1]. Their incidence is typically associated with hypoxia, metabolic disturbances, embolic events, central nervous system infections, or perioperative stroke [2]. While these account for the majority of cases, in endemic regions, such as India, neurocysticercosis (NCC) remains one of the most frequent causes of seizures overall, at approximately 30% [3]. However, the incidental discovery of NCC in the setting of new-onset seizures after cardiac surgery is exceedingly rare and may be overlooked in the acute postoperative phase [4]. Such cases highlight the importance of maintaining a broad differential diagnosis, ensuring that uncommon but clinically significant etiologies like NCC are considered, particularly in patients from endemic areas.

## Case presentation

A 39-year-old woman presented with progressive exertional dyspnea and was diagnosed with severe mitral regurgitation. She was scheduled for elective mitral valve repair with De Vega annuloplasty under general anesthesia. The total cardio bypass time was two hours, and the perioperative period was uneventful.

In the operating theatre, standard American Society of Anesthesiologists (ASA) monitoring (pulse oximetry, ECG, non-invasive blood pressure, and temperature) was instituted. Anesthesia was induced with fentanyl, propofol, and vecuronium, and the trachea was intubated with a size 7 endotracheal tube. Vascular access included a left femoral arterial line and a right internal jugular central venous catheter. Anesthesia was maintained with fentanyl, midazolam, vecuronium, and sevoflurane. The intraoperative course was uneventful, including cardiopulmonary bypass and subsequent weaning. Hemodynamics remained stable throughout.

Postoperatively, the patient was transferred to the intensive care unit (ICU) for elective mechanical ventilation. She was extubated three hours later after meeting standard criteria and maintained stable oxygenation on supplemental oxygen via face mask at 5 L/min.

**Figure 1:**
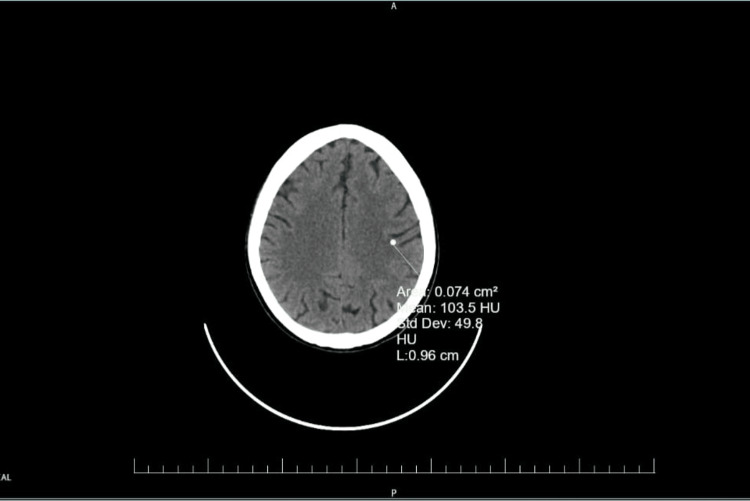
Axial image of non-contrast computed tomography at the level of the parietal region of the brain showing a calcified lesion in the left parietal lobe, consistent with old neurocysticercosis

In the early hours of the following morning, she developed a sudden focal seizure involving clonic movements of the shoulder, which progressed to a generalized tonic-clonic seizure. The episode was self-limiting and controlled with 2 mg intravenous midazolam. Arterial blood gas and serum electrolytes were normal. Four hours later, she experienced another similar seizure, again managed with midazolam. Given the recurrence, urgent neuroimaging was performed. Non-contrast computed tomography of the brain revealed a parenchymal lesion in the left parietal lobe with no perilesional edemasuggestive of a calcified lesion, consistent with old neurocysticercosis (Figure 1). The patient had no prior history of seizures or neurological complaints.

She received an intravenous loading dose of phenytoin, after which no further seizures occurred. Supportive care included close neurological observation, hemodynamic stabilization, and correction of metabolic parameters. Her subsequent recovery was uneventful, and she was mobilized and discharged in stable condition on oral antiepileptic therapy, with detailed advice regarding follow-up for seizure management.

## Discussion

Seizures after cardiac surgery are uncommon but clinically important. Their etiology is multifactorial and includes structural, vascular, metabolic, toxic, and infectious causes. Well-recognized precipitants in the early postoperative period include embolic or hypoperfusion-related stroke, cerebral microembolization during cardiopulmonary bypass (CPB), hypoxia-ischemia, electrolyte or glucose disturbances, renal or hepatic dysfunction, and drug toxicities (such as those from tranexamic acid or certain antibiotics). Contemporary reviews emphasize that although overall seizure incidence is low, the causes are heterogeneous and management must be individualized [5].

In endemic regions, NCC represents an important but underrecognized contributor to seizures. It is the leading cause of acquired epilepsy across much of the Indian subcontinent and other developing regions [3]. Even calcified parenchymal NCC, often regarded as the inactive end stage of infection, can remain epileptogenic for years. Such lesions may trigger focal seizures or focal-to-generalized seizures, sometimes after long seizure-free intervals [6].

In the perioperative setting, various stressors can lower seizure threshold in patients with pre-existing epileptogenic substrates such as calcified NCC. Fluctuations in arterial blood gases, blood pressure changes, microembolic load during CPB, electrolyte derangements, hypoglycaemia, and exposure to proconvulsant medications may all serve as facilitators [7].

Neuroimaging plays a pivotal role in evaluation. Non-contrast CT (NCCT) is often the first-line modality, as it rapidly detects calcified lesions typical of chronic NCC while also excluding acute hemorrhage. In the present case, NCCT revealed an old calcified parietal lesion consistent with NCC, explaining the seizure activity in the absence of acute pathology. Seizure control was promptly achieved with 15 mg/kg intravenous phenytoin at the rate of 50 mg/minute along with supportive optimization of metabolic and hemodynamic parameters as per the Infectious Diseases Society of America (IDSA) [8].

Management priorities in the ICU include rapid assessment of reversible precipitants (glucose, electrolytes, oxygenation, ventilation), immediate seizure control with antiepileptic loading, and close neurological monitoring. Follow-up with neurology is essential to guide long-term antiepileptic therapy and patient counselling regarding recurrence risk. For institutions serving endemic populations, incorporation of NCC into seizure checklists and postoperative protocols may aid timely recognition [9].

The role of routine preoperative neuro-evaluation in cardiac surgical candidates from endemic areas remains uncertain. However, selective screening based on a history of seizures, focal neurological symptoms, or prior neuroimaging abnormalities, along with a low threshold for postoperative imaging when seizures occur, is a pragmatic approach.

## Conclusions

Postoperative seizures following cardiac surgery are rare but clinically significant, requiring a wide differential diagnosis that includes vascular, metabolic, hypoxic, infectious, and drug-related causes. In endemic regions, neurocysticercosis must also be considered, even when there is no prior neurological history. Calcified NCC lesions, though often considered inactive, can remain epileptogenic and may become symptomatic in the physiologically stressful postoperative period.

This case underscores the importance of maintaining a high index of suspicion for atypical causes of seizures, particularly in patients from endemic areas. Timely neuroimaging, rapid initiation of antiepileptic therapy, and supportive management are critical for favourable outcomes. Greater awareness among anesthesiologists, intensivists, and surgeons can help reduce the morbidity associated with such unexpected neurological complications.
